# Cyber-intimate partner violence (C-IPV) and its risk factors among women in Malaysia

**DOI:** 10.1371/journal.pone.0327978

**Published:** 2025-07-30

**Authors:** Yuhaniz Ahmad, Salmi Razali

**Affiliations:** 1 Department of Psychiatry, Faculty of Medicine, Hospital Al-Sultan Abdullah, Universiti Teknologi MARA, Selangor, Malaysia; 2 School of Quantitative Sciences, Universiti Utara Malaysia, UUM Sintok Kedah, Malaysia; The Hong Kong Polytechnic University, HONG KONG

## Abstract

Cyber Intimate Partner Violence (C-IPV) is a serious challenge that negatively impacts the physical, mental and social health of victims. This study aims to estimate the prevalence of C-IPV among Malaysian women and identify associated risk factors. This cross-sectional study used an online questionnaire distributed through social media. A total of 1,838 female respondents aged 18–62 years participated, with 49.6% reported experiencing C-IPV. By taking into account all variables into a multiple logistic regression model, significant risk and protective factors for C-IPV were identified. Ethnicity, occupation, income and alcohol consumption were significantly increased the odds of C-IPV, while religion, marital status and number of active partners were identified as significant protective factors. The results of the study showed a significant positive relationship between C-IPV and face-to-face intimate partner violence (F2F-IPV). These findings emphasize the need for holistic intervention strategies involving multiple parties to eliminate intimate partner cyberbullying. This study suggests improving cybersecurity policies and community education programs to increase awareness and prevention of C-IPV.

## Introduction

Intimate partner violence (IPV) and cyberbullying are public concerns that result in various detrimental consequences [1]. The United Nations defines cyberbullying as repetitive behaviours that use digital technologies via social media, messaging platforms, gaming platforms or mobile phones with the intention to scare, anger and shame the targeted victims [2]. According to Module 12 of Interpersonal Cybercrime by the United Nations Office on Drugs and Crime, interpersonal gender-based violence, which includes IPV, can be committed offline or online [3]. Cyberbullying behaviours that occur within the context of intimate relationships can be considered as a digital extension of traditional IPV, which is also known as Cyber-IPV or C-IPV.

The latest report by the World Health Organization (WHO), estimates that the highest lifetime prevalence of Face-to-Face IPV (F2F-IPV) ranges from 40% to 53% were recorded in 19 countries, occurring among women aged 15–49 years. The overall prevalence rate is 31%, or 1 in 3 women have experienced at least one form of violence in their lifetime [4]. As for cyberbullying, in 2020, the Office for National Statistics estimated that the prevalence of cyberbullying in the United Kingdom was around 28% among disabled people and 18% among those who were not [5]. In New Zealand, a 2019 report estimated the prevalence of cyberbullying among adults to be approximately 14.9%, and about 2.2% reported experiences of cyberbullying within the previous 30 days [6]. A recent online survey of 1449 participants from Selangor, Malaysia, indicates a slightly higher percentage of 1-month prevalence (2.4%) of cyberbullying victimisation as in New Zealand [7]. In terms of C-IPV, according to a study conducted in high-income countries in North America and Europe, about 85.7% of female victims of cyberbullying were also classified as victims of IPV, with a higher prevalence (95.2%) among younger women aged 18–24 years [8].

Cyberbullying is a modern form of bullying, such as hacking, stalking, impersonation, spreading false rumours, sending hostile or threatening messages, making harassing phone calls, or sharing private photos or videos without consent [9]. Cyberbullying provides an easy opportunity for aggression and violence, such as embarrassing, hurting or degrading others through cyber-media such as *Facebook, YouTube, WhatsApp, TikTok* and many others [10]. Cyberbullying can quickly go viral, making it a highly public and embarrassing experience for the victims. The perpetrators can post humiliating videos and pictures, lies or insulting language on social networks, track locations via GPS and send threatening messages directly to the victim anytime and anywhere [11]. Previous studies in the United Kingdom revealed that often the cyberbullying victims and the perpetrators know each other. In addition, out of revenge motives, these perpetrators spread sexual material that is related to the victim [12]. Moreover, given the wide distribution and sustainable storage of digital technologies, deleting the messages or degrading videos that have been spread is not an easy task [13]. Through digital technology, C-IPV can be accessible at any time, and its perpetrators can remain anonymous, making it more challenging for victims to defend themselves.

C-IPV has been linked to increased social pressure, adverse physical health and elevated psychological distress [14]. Family dysfunction and low self-esteem were significant risk factors for cyberbullying victimization. Meanwhile, frequent internet use and low empathy have been associated with increased psychological distress [7]. C-IPV is often used as a means of asserting control over a partner even when they are not physically present. Women who experience IPV are at significantly higher risk of developing mental health disorders, including depression and suicidal ideation, compared to those who do not experience such violence [15]. Bullying and IPV are characterized by an imbalance of power within interpersonal relationships, which may lead aggressors who engage in bullying to carry over such abusive behaviours into their in-person romantic partnerships. Beyond mental health impacts, C-IPV can also have economic and social consequences. Victims of C-IPV often face financial instability, job insecurity, and difficulty maintaining stable housing [15].

Despite the dangerous and detrimental consequences of C-IPV, in Malaysia, sparse knowledge on the prevalence of C-IPV and its associated factors to inform effective interventions to address these duo phenomena. Understanding the relationship between these forms of violence is crucial for developing effective interventions and policies to eradicate them. Therefore, this study aims to estimate the prevalence of C-IPV among the Malaysian female population and determine the risk factors of C-IPV victimisation among them.

## Materials and methods

### Study design

This is a cross-sectional online study on Malaysian women to determine the prevalence of C-IPV and their relationship with socio-demographic factors, smoking and alcohol consumption, as well as details cyberbullying behaviours. Given the nature of C-IPV, which often occurs in covert environments [16], an online survey is used to improve access to this target population.

### Data collection

Data was collected through a convenience sampling technique. The survey was distributed to the public through Google Forms, which was shared on social media platforms including *Facebook, Instagram, Twitter, and WhatsApp*. The inclusion criteria were participants aged 18 years or older, proficient in the Malay language, in an intimate relationship, and having access to the internet to complete the online questionnaires. Participants were informed of the risks and benefits of the study on the introductory page. Those who agreed to participate were invited to complete the questionnaires. Implied consent was obtained when participants proceeded to the next page of the online survey, in accordance with procedures approved by the UiTM Research Ethics Committee. Data collection was done from July 2023 to May 2024.

### Measurement tools

We utilized a structured questionnaire written in Malay, comprising three sections: socio-demographic background, the Malay Version-Women Abuse Screening Tool (WAST) for screening IPV victims, and the Cyberbullying Victimization Scale (CVS) to document cyber-specific abuse, allowing a clear separation between F2F-IPV and C-IPV.

The initial section of the questionnaire captured socio-demographic information, including gender, ethnicity, employment, marital status, and total household income. In Malaysia, socioeconomic status is categorized into three groups: B40 (total household income less than RM4,849), M40 (total household income between RM4,850 and RM10,959), and T20 (total household income exceeding RM10,959). This section also included questions about behaviours related to substance use, such as smoking, alcohol consumption, and drug use.

The Malay Version-WAST has cut-off scores equal to 15 and acceptable Cronbach’s alpha (0.873), sensitivity (0.596) and specificity (0.767) [17]. The first two WAST questions offered three response options: 1 (no tension/no difficulty), 2 (some tension/some difficulty) and 3 (a lot of tension/great difficulty). The remaining six WAST questions also used a 3-point scale ranging from 1 (never) to 3 (often), yielding a total score range of 8–24.

The CVS questions have a Cronbach’s alpha of 0.92, indicating high internal consistency. This scale included nine items rated on a scale from 0 (never) to 3 (many times), generating a continuous variable that reflects the overall types and frequency of cyber-victimization experiences. Higher total scores indicate more frequent or severe cyber-victimization. Cyberbullying was considered present if respondents reported experiencing a specific type of cyberbullying more than once [8].

### Data analysis

The Chi-squared analysis (χ2) was then applied to determine the associations between IPV and socio-demographic parameters and the 9 items of CVS. To estimate the adjusted odds ratios for C-IPV victimization based on demographic characteristics and cyberbullying experiences, all socio-demographic parameters that demonstrated significant associations with C-IPV in the Chi-squared analysis were included in a multiple logistic regression model.

### Ethical considerations

This study was approved by the Universiti Teknologi MARA research ethics committee in June 2023; REC/06/2023(PG/FB/12).

## Results

### Background participants

A total of 1838 female participants answered the survey. The participants were predominantly Malay (1002;54.5%), Muslims (1011;55.0%) and married (1578;85.9%) women. The majority of participants were among those in a middle group of socioeconomic status (M40) (1339;72.9%), have at least secondary education (1039;56.5%), working (1554;89.4%) and mostly have only one partner (1191;64.8%).

### Face-to-face IPV (F2F-IPV) and socio-demography

A total of 1163(63.3%) of the participants have experienced F2F-IPV. Of the total women who have F2F-IPV, they were Muslims (599; 51.5%), married (1060; 91.1%), working (1040;89.4%), had secondary education (724; 62.3%), came from M40 group (924; 79.4%), never had an intimate relationship with more than one person (721: 62%), non-smoker (534; 45.9%) and never consume alcohol drink (516; 45.4%). F2F-IPV are significantly (*p* < 0.05) associated with religion, ethnicity, marital status, working status, education level, family income, smoking, alcohol intake and number of active partner(s) at a time (Table 1).

**Table 1:** Crosstabulation of the respondents for F2F-IPV status versus Sociodemographic.

		Face-to-Face IPV, n (%)			Statistical Analysis	
Sociodemographic data		No (n = 675)	Yes (n = 1163)	Total (n = 1838)	$\mathrm{\backslash bold}\chi^{2}$	*p*
Religion	Islam	412(61.0)	**599(51.5)**	1011(55.0)	24.727	**0.000** ^*^
	Hindu	67(9.9)	123(10.6)	190(10.3)		
	Christian	128(19.0)	239(20.6)	367(20.0)		
	Others	4(0.6)	5(0.4)	9(0.5)		
	No Religion	64(9.5)	197(16.9)	261(14.2)		
Ethnic	Malay	401(59.4)	**601(51.7)**	1002(54.5)	28.263	**0.000** ^*^
	Chinese	173(25.6)	422(36.3)	595(32.4)		
	Indian	75(11.1)	120(10.3)	120(10.3)		
	Sabah Bumiputera	12(1.8)	11(0.9)	11(0.9)		
	Sarawak Bumiputera	8(1.2)	6(0.5)	6(0.5)		
	others	6(0.9)	3(0.3)	3(0.3)		
Marital status	Single	60(8.69)	26(2.2)	86(4.7)	82.243	**0.000** ^*^
	Married	518(76.7)	**1060(91.1)**	1578(85.9)		
	Widow/Widower	59(8.7)	47(4.0)	106(5.8)		
	Separated	20(3.0)	23(2.0)	43(2.3)		
	Divorce	18(2.7)	7(0.6)	25(1.4)		
Employment Status	Working	514(76.1)	**1040(89.4)**	1554(89.4)	62.269	**0.000** ^*^
	Not working	73(10.8)	68(5.8)	68(5.8)		
	Retired	35(5.2)	28(2.4)	28(2.4)		
	Study	53(7.9)	27(2.3)	27(2.3)		
Education level	Primary	61(9.0)	46(4.0)	107(5.8)	50.462	**0.000** ^*^
	Secondary	315(46.7)	**724(62.3)**	1039(56.5)		
	Tertiary Education	294(43.6)	383(32.9)	677(36.8)		
	No education	5(0.7)	10(0.9)	15(0.8)		
Family Monthly Income	B40	204(30.2)	179(15.4)	383(20.8)	70.673	**0.000** ^*^
	M40	415(61.5)	**924(79.4)**	1339(72.9)		
	T20	56(8.3)	60(5.2)	116(6.3)		
Number of Partners at a time	1	470(69.6)	**721(62.0)**	1191(64.8)	13.031	**0.011** ^*^
	2	124(18.4)	244(21.0)	368(20.0)		
	3	57(8.4)	149(12.8)	206(11.2)		
	4	17(2.5)	35(3.0)	52(2.8)		
	5 or more	7(1.0)	14(1.2)	21(1.1)		
Smoking status	Not smoking	364(53.9)	**534(45.9)**	898(48.9)	18.033	**0.000** ^*^
	Not everyday	240(36.5)	531(45.7)	771(41.9)		
	Everyday	71(10.5)	98(8.4)	169(9.2)		
Alcohol intake	Never	364(53.9)	**516(44.4)**	880(47.9)	75.358	**0.000** ^*^
	More than 1 year ago	77(11.4)	100(8.6)	177(9.6)		
	1-2 times a year	104(15.4)	156(13.4)	260(14.1)		
	1-3 times a month	57(8.4)	88(7.6)	145(7.9)		
	1-2 times a week	51(7.6)	273(23.5)	324(17.6)		
	Almost everyday	22(3.3)	30(2.6)	52(2.8)		

Notes: χ^2^ = Chi-Squared test;

* The test is significant with *p* < 0.05

### Cyberbullying and socio-demography

The prevalence of cyberbullying among women in the sample is 76.7% (n = 1409). Among those who experienced cyberbullying, they have had someone posted online about them in the form of a bad or hurtful comment (921;65.4%), picture (921;65.4%), video (884;62.7%), nasty or hurtful webpage (884;62.7%) and rumours (903;64.1%); and threatened to hurt them through a cell phone text message (899;63.8%) and online (913;64.8%) and has had someone pretended to be them online and acted in a mean or hurtful way (896;63.6%).

### Cyber-Intimate Partner Violence (C-IPV)

Of the total 1838 female respondents (mean age of 32.89 ± SD 9.396 and range 18–62 years), 912 (49.6%) respondents reported experiencing both F2F-IPV and any type of cyberbullying more than once. When asked in detail regarding the cyberbullying sub-items, of the total of 1163 respondents with F2F-IPV, 648 (55.7%) shared that they have been cyberbullied. Sub-analysis of the correlation between C-IPV and F2F-IPV yields a significantly strong positive correlation between both violent behaviours (*r* = 0.756; *p* < 0.001). This strong correlation between C-IPV and F2F-IPV suggests that these two forms of violence may be closely related, such as having similar patterns or underlying causes. The sub-analyses of each item of CVS indicated that the F2F-IPV have had someone posted online about them in the form of a bad or hurtful comment (613; 52.7%), picture (610; 52.5%), video (582; 50.0%), nasty or hurtful webpage (577; 49.6%) and rumours (588; 50.6%); and threatened to hurt them through a cell phone text message (590; 50.7%) and online (596; 51.2%) and has had someone pretended to be them online and acted in a mean or hurtful way (573; 49.3%). Only three types of C-IPV are significantly related to F2F-IPV, which are those who experienced a bad comment or hurtful comment ($\chi^{2}$=7.188; *p* = 0.007), a bad or hurtful picture posted (χ^2^ = 6.946; *p* = 0.008) and threatened to hurt through cell phone text message (χ^2^ = 4.193; *p* = 0.041) (Table 2).

**Table 2:** Associations between IPV and cyberbullying.

Cyberbullying		Face-to-Face IPV victims, n (%)			Statistical Analysis	
		No (n = 675)	Yes (n = 1163)	Total	Cochran’s $\chi^{2}$	*p*
Experienced any type of cyberbullying more than once.	No	178(26.4)	251(21.6)	429(23.3)	5.473	**0.019** ^*^
	Yes	497(73.6)	**912(78.4)**	1409(76.7)		
Ever been a victim of cyberbullying (CVS1)	No	328(48.6)	515(44.3)	843(459)	3.196	0.074
	Yes	347(51.4)	**648(55.7)**	995(54.1)		
Sub-analyses of associations between IPV and Cyberbullying						
Someone ever posted a bad comment about you or that hurt online (CVS2)	No	363(53.8)	550(47.3)	913(49.7)	7.188	**0.007** ^*^
	Yes	312(46.2)	**613(52.7)**	925(50.3)		
Someone ever posted a bad or hurtful picture of you online (CVS3)	No	364(53.9)	553(47.5)	917(49.9)	6.946	**0.008** ^*^
	Yes	311(46.1)	**610(52.5)**	921(50.1)		
Someone ever posted a bad or hurtful video of you online (CVS4)	No	369(54.7)	581(50.0)	950(51.7)	3.794	0.051
	Yes	306(45.3)	**582(50.0)**	888(48.3)		
Someone ever created a nasty or hurtful web page about you (CVS5)	No	366(54.2)	586(50.4)	952(51.8)	2.516	0.113
	Yes	309(45.8)	**577(49.6)**	886(48.2)		
Someone spread rumours about you online (CVS6)	No	360(53.3)	575(49.4)	935(50.9)	2.589	0.108
	Yes	315(46.7)	**588(50.6)**	903(49.1)		
Someone ever threatened to hurt you through a cell phone text message (CVS7)	No	366(54.2)	573(49.3)	939(51.1)	4.193	**0.041** ^*^
	Yes	309(45.8)	**590(50.7)**	899(48.8)		
Someone ever threatened to hurt you online (CVS8)	No	358(53.0)	567(48.8)	925(50.3)	3.135	0.077
	Yes	317(47.0)	**596(51.2)**	913(49.7)		
Someone pretended to be you online and acted in a mean or hurtful way (CVS9)	No	351(52.0)	590(50.7)	941(51.2)	0.275	0.600
	Yes	324(48.0)	**573(49.3)**	897(48.8)		

Notes: χ^2^ = Cochran’s’ Chi-Squared test;

* The test is significant with *p* < 0.05

### Risk factors for C-IPV

Multiple logistic regression indicates that significant risk factors for C-IPV are ethnicity (OR=4.868), employment (OR=2.154), income (OR=1.304) and alcohol consumption (OR=1.733). The protective factors of C-IPV include religion (OR=0.113), marital status (OR=0.511) and number of active partners (OR=0.525) (Table 3).

**Table 3:** Multiple Logistic Regression of socio-demographic factors and cyber-IPV status.

	$\hat{\beta }$	S.E.	Sig.	Odds Ratio (OR)	95% C.I. for Exp ($\hat{\beta }$)	
					Lower	Upper
Religion	−2.178	0.767	**0.005** ^*^	0.113	0.025	0.510
Ethnic	1.583	0.766	**0.039** ^*^	4.868	1.085	21.854
Education	0.029	0.108	0.791	1.029	0.832	1.273
Employment	0.767	0.159	**0.000** ^*^	2.154	1.579	2.939
Income	0.266	0.128	**0.038** ^*^	1.304	1.015	1.676
Marital status	−0.672	0.266	**0.012** ^*^	0.511	0.303	0.861
Number of active partners	−0.645	0.118	**0.000** ^*^	0.525	0.416	0.662
Smoking	0.161	0.150	0.283	1.174	0.876	1.574
Alcohol	0.550	0.163	**0.001** ^*^	1.733	1.258	2.388
Constant	−0.463	0.214	**0.031** ^*^	0.629	0.025	0.510

Items in the table refer to Muslim, Malay, secondary school of education, working, income in the middle 40% of the population, married, has only one active partner at a time, smoking and consuming alcohol.

* The estimate is significant at α=5%.

## Discussion

Our study indicated that among females aged 18–62, the prevalence of C-IPV is 49.6%. This prevalence is within the percentages of C-IPV as described by the meta-analysis studies of the majority of countries worldwide [14]. After adjusting the confounding factors using multiple logistic regression, we found that ethnicity, employment, income and alcohol consumption are the significant risk factors for C-IPV, while religion, marital status and number of active partners are the protective factors.

Being Malay (the dominant ethnicity in Malaysia) increases the odds of women aged 18–62 years becoming victims of C-IPV by approximately fivefold (Adjusted Odds Ratio [AOR] = 4.868; 95% CI: 1.085–21.854). This finding contradicts a previous study conducted in Malaysia, which reported that Chinese women were more frequently the victims of cybercrime [18]. Elsewhere, a study in another country such as Baltimore where Black or African Americans make up 60.7% of the population, also indicated the presence of a high prevalence of IPV among the dominant ethnic group in that country [19]. Perhaps, the high prevalence of Malays in cases of F2F-IPV correlates with the increase in C-IPV among this ethnic group too. Positive large effect sizes for the correlation between C-IPV and F2F-IPV perpetration have been described by other studies [14]. Although the majority of Malay participants—most of whom identified as Muslim—were found to be at heightened risk of experiencing cyber-intimate partner violence (C-IPV), religious affiliation, particularly adherence to Islamic principles, appears to function as a protective factor. This suggests that the association between religion and C-IPV risk is likely moderated by other cultural or contextual variables. Therefore, it is essential to promote the genuine internalization and practice of Islamic teachings, which categorically prohibit all forms of harm, including cyberbullying and intimate partner violence, as these values are central to the effective prevention of C-IPV [20]. Certainly, path analysis, which can examine the effect of moderation between these two variables, is suggested for future study.

In this study, employment and income are two important socioeconomic indicators that increase the odds of becoming the victim of C-IPV. Working women have about twice the odds, and those in the M40 group have 1.3 odds of becoming the victims of C-IPV respectively. One possible explanation for these findings is that stable jobs and financial security provide women in these groups with greater access to digital technologies such as mobile phones, computers or other gadgets which may increase their exposure to C-IPV. This hypothesis warrants further investigation. More in-depth discussion through resource theory [21] suggested that men may result in IPV perpetration where other resources to establish power and control are lacking. Hence, in C-IPV when men have lower economic status than women, they cannot exert power in the real world because of financial limitations, and direct the power and control through cyber-media [22]. Moreover, a study in Egypt suggested that education and employment continue to be among the main factors for IPV, but only specific to physical and psychological IPV, not for other types of violence [23]. Interestingly, gendered resource theory (when traditional power dynamics have been disrupted, such as women becoming financially independent, men may exert control through IPV), patriarchal theory (male dominance is a structural cause of IPV) and empowerment theory (increasing women’s autonomy initially triggers resistance from partners) are suggested to be able to moderate those former theories [22].

There is an increasing alcohol, drug and other substance use behaviour among young people including young women in Malaysia [24]. Studies elsewhere have shown that those behaviours correlate with F2F-IPV and C-IPV [14]. Local researchers also highlighted that females who use substances are vulnerable to IPV [25]. In their study with 200 females who use substances in a rehabilitation centre, the prevalence of IPV among them was 53%, a much higher percentage compared to those who did not use substances. Other factors associated with this group of young females were living away from parents or family, having a regular intimate male partner who also uses drugs, and having been a victim of underage rape [25].

We also found that being married and having only one active partner are protective factors for C-IPV. In other words, a person’s chances of experiencing C-IPV increase when they are unmarried and have more than one active partner. The lack of formal commitment in a relationship leads to emotional instability, lack of trust, and more frequent conflict, leading to controlling or manipulative behaviours through technology. Additionally, relationships with more than one active partner may lead to jealousy and competition, resulting in violent behaviours or excessive monitoring [26–28].

## Conclusion

This study’s findings have to be understood with few limitations. This study used convenience sampling, which is cost-effective and quick, but limits generalizability. Its cross-sectional design provides a snapshot of prevalence and associated factors but cannot establish causation or a comprehensive picture over time. While this study provides a valuable snapshot of the prevalence and correlates of C-IPV, future research using longitudinal or mixed-method designs is recommended to establish causal relationships and deepen contextual understanding. Additionally, reliance on self-reported data introduces potential response bias, as participants may underreport or overreport their experiences due to stigma, recall issues, or social desirability. Furthermore, we acknowledge that the recruitment process was done via a social media platform, which poses challenges such as sampling bias, limited demographic reach, privacy concerns, and difficulty verifying participant identity. Platform restrictions and inconsistent visibility further complicate recruitment, potentially impacting data validity and the overall reliability of research findings.

This study contributes to the understanding of the significant relationship between socio-demographic background (such as ethnicity, employment, income, religion, marital status and number of active partners as well as alcohol consumption) and C-IPV. It is crucial for various stakeholders, including policymakers, agencies related to cyber-security, law enforcement, healthcare professionals, and community organizations to address this detrimental behaviour urgently. The advances in the digital era should be in keeping with the increase in the safety of the cyber-world and eradication of any type of violence, including C-IPV.
